# Factors associated with food label use: focus on healthy aspects of orthorexia and orthorexia nervosa

**DOI:** 10.1007/s40519-024-01661-9

**Published:** 2024-05-04

**Authors:** Ezgi Bellikci-Koyu, Yasemin Karaağaç, Armağan Aytuğ Yürük

**Affiliations:** 1 Department of Nutrition and Dietetics, Faculty of Health Sciences, Izmir Kâtip Çelebi University, Izmir, Türkiye; 2 Department of Nutrition and Dietetics, Faculty of Health Sciences, Erciyes University, Kayseri, Türkiye

**Keywords:** Nutrition facts panel, Ingredients list, Claims, Orthorexia, Food label knowledge

## Abstract

**Purpose:**

This study aimed to investigate the potential relationships between the use of different section of food label, and healthy and pathological aspects of orthorexia among adults.

**Methods:**

This cross-sectional study was conducted using an online survey (*n* = 1326). Inclusion criteria were being 19–64 years and graduated from at least primary school. Pregnant and lactating women were excluded. Data were collected using questionnaire including socio-demographic variables, lifestyle factors, body weight and height, frequency of reading different sections of food label (“always”, “when buying a food for the first time”, “when comparing similar packaged foods”, “rarely”, “never”), food label literacy, and Teruel Orthorexia Scale. Participants were categorized as nutrition facts panel-users, ingredients list-users or claim-users if they read at least one item from the relevant parts.

**Results:**

The proportions of nutrition facts, ingredients list, and claims sections users were 72.3%, 76.3%, and 79.9%, respectively. Both healthy and pathological aspects of orthorexia were associated with reading food labels. The healthy orthorexia had the strongest association with using the ingredients list (OR 1.76, 95% CI 1.41–2.20), whereas the orthorexia nervosa showed the highest association with using nutrition facts panel (OR 1.48, 95% CI 1.20–1.81). While women, physically active participants and those with higher food label literacy were more likely to use all sections of food labels; older age, having children, and chronic disease increased the likelihood of using claims and ingredients list (*p* < 0.05). Besides, following a diet was associated with higher use of nutrition facts and ingredients list (*p* < 0.05).

**Conclusions:**

The study demonstrates that food label users have higher orthorexia tendencies compared to non-users. Of the food label sections, healthy orthorexia showed the strongest association with use of the list of ingredients, while pathological orthorexia showed the strongest association with use of the nutrition facts panel.

**Level of evidence:**

Level V, cross-sectional study.

**Supplementary Information:**

The online version contains supplementary material available at 10.1007/s40519-024-01661-9.

## Introduction

In recent decades, all countries have experienced a nutrition transition, which refers to a change from traditional diets towards a more processed global diet that is closely connected to the industrialization and globalization of the food production and distribution systems [1–3]. In parallel with changes in nutrition, various dietary guidelines [4–7] and organizations [8–10] have recommended the use of food labels as a tool to help consumers make healthier food choices. The food labels are defined as any written, printed, or graphic information on or attached to a food product container, providing details about the product’s identity and contents, and on how to prepare and consume it safely [8]. Specifically, the mandatory “Nutrition Facts Panel” and “Ingredients List” section of the food label, together with the voluntary “Claims” section, provide valuable information to help consumers make healthy food choices.

Consumer characteristics such as being female [11–14], having higher levels of education [11–14] and income [11, 13], being physically active [11] having chronic disease [12] and weight goal [11] are considered important factors associated with a higher likelihood of using food labels. However, the relationship between the use of food labels and age is conflicting [11, 12, 14]. Nutrition knowledge of the consumer is also a crucial factor [15], as it affects how they perceive and understand the information on food packaging. Several studies also show that nutritional [16–20] and, in particular, food label knowledge [21] are related to the use of food labels. However, studies investigating the relationship between reading different sections of food labels and consumer characteristics are scarce. To our knowledge, only one study has been conducted to directly assess the frequency and characteristics of those who read different sections of food labels [22]. This large-scale study revealed that the nutrition facts panel was used by 61.6% of participants, the list of ingredients by 51.6%, the serving size by 47.2%, and health claims by 43.8% at least sometimes when deciding to purchase a food product. It also reported that women, older age groups and adults with higher levels of education and income were more likely to read all sections of the food label. However, the study only investigated basic socio-demographic characteristics and did not include characteristics such as health status, level of physical activity, dieting status, obsession with healthy eating, or knowledge about nutrition or food labels, which are known to be associated with food label use. Therefore, there is a need to further investigate the relationship between reading different parts of the food label and consumer characteristics.

Consumers' attempts to maintain a healthy diet are significantly related to the use of food labels [23, 24]. In this context, orthorexia, derived from the Greek words 'ortho' and 'orexis' and means 'right appetite' [25], has been linked to the reading food label. To our knowledge, only one study has investigated the food label reading habits among university students with and without tendencies toward orthorexia nervosa [26]. The study reported that individuals with tendencies towards orthorexia nervosa have a higher frequency of reading food labels compared to individuals with normal eating behaviour. However, in that study, orthorexia was only evaluated from its pathological aspect. But, according to the literature, orthorexic behaviour can manifest as a reasonable concern for proper nutrition or a compulsive fixation on healthy eating. Although Orthorexia Nervosa, which is a pathological aspect of orthorexia, is not recognized as a distinct eating disorder in the International Classification of Diseases-11 (ICD-11) or the Diagnostic and Statistical Manual of Mental Disorders-5 (DSM-5), it is typically defined as an obsessive preoccupation with healthy food choices, leading to restrictive dietary rules that can cause emotional distress (which may progress to physiological dysfunction) and orthorexia nervosa make the individual more stressed about eating [27, 28]. To differentiate non-pathological thinking about healthy eating from orthorexia nervosa disorder, another term, "healthy orthorexia" has begun to be used [29, 30]. The most used self-report measure to assess orthorexia nervosa is the ORTO-15 [28]. However, it has demonstrated an unstable factorial structure across different populations and has been criticized for its poor psychometric quality, and inability to distinguish between healthy and pathological eating [28, 31]. A relatively new scale, Teruel Orthorexia Scale, has been developed to evaluate both healthy orthorexia and orthorexia nervosa [25].

Studies point out the difficulty of distinguishing between orthorexia nervosa and healthy orthorexia because they share similar behavioural attitudes [25, 28, 30], but also emphasize that they differ in some behaviours [25, 30]. To diagnose orthorexia nervosa accurately, it is crucial to identify the behaviours and characteristics that differentiate between pathological and healthy aspects of orthorexia. Therefore, investigating whether these two orthorexic behaviours show different tendencies in terms of food label reading, which is critical for healthy food choices, would be of interest. Identifying the relationships between orthorexic behaviours and food label reading habits, and the parts of the food label that are focused on, may help to distinguish the characteristics of two different aspects of orthorexia and to establish diagnostic criteria for orthorexia nervosa. Considering this background, the main objective of this study is to examine the potential relationship between the use of different sections of food labels and both aspects of the orthorexic tendency among adults, as they represent the largest proportion of society and play a crucial role in food purchasing. Secondary objective is to evaluate the frequency of food label use and consumer characteristics associated with its usage.

## Materials and methods

### Participants and procedures

This descriptive and cross-sectional research was conducted from November 2020 to January 2022. An online questionnaire was carried out and potential participants were provided with the link to the questionnaire. The snowball sampling technique was used, and it was initiated from multiple sources both the students mentioned in the “Acknowledgements” section and the authors of this article. Initially, the survey link was shared on several social media platforms (WhatsApp, Instagram, Facebook, and Telegram groups), and participants were recruited from these various social media groups. These initial participants were then requested to further distribute the survey among their contacts and social media accounts to expand the reach of the study. This approach was chosen to take advantage of the network effect and extend our reach beyond the initial connections. Throughout the study period, the survey link was regularly shared on social media accounts at least once a month. Inclusion criteria required participants to be aged between 19 and 64 and to have completed primary school or equivalent. Pregnant and lactating women were excluded. Before the research began, the survey was distributed to 28 participants, and modifications were carried out in response to the feedback received. These comments were related to the lack of understanding of the terms such as sodium, nutrition claim and health claim. In response, the term sodium was changed to sodium/salt and health and nutrition claims were defined and exemplified in the revised survey. In the study, a total of 1534 responses were received. Among these, 81 were duplicate cases, 45 individuals were excluded due to pregnancy or being in the breastfeeding period, and 82 people were excluded because they did not complete the survey. Thus, the study was completed with a total of 1326 participants.

### Personal information, health status and lifestyle characteristics

Personal information including age, sex, educational level, income, marital and parental status was collected. Self-reported body weight (kg) and height (cm) were obtained, and the body mass index (BMI) was calculated. Participants’ health status and lifestyle characteristics were assessed by asking whether they have a chronic disease, follow a regular diet, have a food allergy, and exercise regularly.

### Orthorexic behaviour

Orthorexic eating behaviour was assessed using Teruel Orthorexia Scale (TOS). TOS was developed by Barrada and Roncero in 2018 [25], and its Turkish reliability and validation were conducted by Asarkaya and Arcan [32]. The scale evaluates orthorexia in two subscales including healthy orthorexia (TOS–HeOr) and orthorexia nervosa (TOS–OrNe). The Turkish version of the scale consists of 16 items (9 items for TOS–HeOr and 7 for items TOS–OrNe) and responses are scored on a 4-point scale from 0 “completely disagree” to 3 “completely agree”. Subscales scores are calculated by summing related items. Higher scores on the TOS–HeOr represent a non-pathological interest and engagement in healthy eating, whereas higher scores on the TOS–OrNe indicate an obsessional or pathological preoccupation with healthy nutrition. Cronbach’s alpha value for TOS–HeOr was 0.86; and it was 0.81 for TOS–OrNe in the Turkish population [32]. In this study, Cronbach’s alpha value was found 0.86 for TOS–HeOr and 0.87 for TOS–OrNe.

### Food label use

The use of three different types of food label information, including nutrition facts, ingredients list, and health and nutrition claims were assessed with 15 items. These items encompassed the information present on food labels in Türkiye. Participants indicated how often they read nutrition facts panel for energy, total fat, saturated fat, protein, carbohydrate, dietary fiber, sugar, sodium, and vitamin–mineral items. The frequency of checking the ingredients list was assessed with following items; ingredients, additives, and allergen content. Lastly, the using frequency of the health claim, nutrition claim, and organic logo was inquired. Response options for all questions were “always”, “when buying a food for the first time”, “when comparing similar packaged foods”, “rarely”, and “never”. Participants were classified as either food label users or non-users, following the approach of Ollberding et al. [22]. Participants who responded by reading “always”, “when buying a food for the first time”, or “when comparing similar packaged foods” at least for one of the items among the nutrition facts panel were considered as nutrition facts panel-users. Other participants were grouped as nutrition facts panel non-users. A similar classification was performed for the ingredients list and claims. In this regard, participants who read at least one of the items among the ingredients list (ingredients, allergen content, and additives) and those checking at least one of the items among claims (health claim, nutrition claim, organic logo) were labelled as ingredients list-user or claims-user, respectively (Supplementary Fig. S1).

### Food label literacy

Food label literacy was assessed by using the last section of the Evaluation Instrument of Nutrition Literacy on Adults (EINLA). The EINLA scale was developed by Cesur et al. in 2015 for the Turkish population to measure nutrition literacy levels [33]. The whole scale consists of 35 questions and five sections. Since the study aimed to determine the literacy of food labels, only the fifth section of EINLA was used. Some previous studies have also conducted separate analyses on the sub-dimensions of EINLA [34–36]. This specific section (composed of 6 questions) focuses on the ability to understand and interpret food labels and perform basic mathematical calculations. While participants received one point for each correct answer, they received zero points for unanswered questions or incorrect answers [33]. In this study, the Cronbach’s alpha coefficient was 0.77 for the fifth section of EINLA.

### Ethical approval

The study protocol was approved by the local ethics committee (Izmir Katip Celebi University Non-Interventional Clinical Studies Institutional Review Board, Decision No:1081, Decision Date: 19.11.2020). This study complies with the Declaration of Helsinki.

### Statistical analyses

Descriptive statistics were presented as numbers and percentages for categorical variables and mean and standard deviations for continuous variables. The normality of the data was evaluated using Skewness and Kurtosis and the values between − 1.5 and + 1.5 were considered normally distributed [37]. While all scales were normally distributed, independent samples *t* test was used for group comparison of the continuous variables. Categorical variables were analysed using the *X*^*2*^ test. Relationships between different areas of food label use and healthy orthorexia and orthorexia nervosa were assessed with binary logistic regression. First, unadjusted odds ratios (ORs) and 95% confidence intervals (CIs) were presented. Afterward, possible factors identified in the univariate analysis were entered into the logistic regression analyses. In the adjusted model 1, age, sex, marital status, having children, having chronic disease, dieting and physical activity were added to the model, and model 2 included food label literacy additionally. Since a single point increase in the total orthorexia scores would not provide a clinically significant evaluation in terms of odds ratio, averaged scores of the scales were used in the logistic regression. All statistical analyses were performed using IBM SPSS Statistics for Windows, Version 25 (IBM Corp., Armonk, New York), and *p* < 0.05 was considered statistically significant.

A posteriori power analyses were separately performed for TOS–HeOR and TOS–OrNe scores based on the use of nutrition facts panel. The power (1-*β*) was determined as 0.99 for TOS–HeOR and 0.89 for the TOS–OrNe scores. The power calculation was performed with G*Power software (Version 3.1. 9.7).

## Results

Among 1326 participants, 67.1% were women, and the mean age was 37.82 ± 14.07 years (range 19–64). The majority of participants (79.3%) had a university or higher degree, and almost half of them had children. Approximately one-third of the participants had a chronic disease, 12.6% were dieting, and 35.1% were doing regular physical activity (Table 1). The mean BMI was 24.63 ± 4.65 kg/m^2^, 30.9% of the participants were overweight and 12.0% had obesity.

**Table 1:** Descriptive statistics of the participants (*n* = 1326)

Characteristic	*n*	%
Sex		
Men	436	32.9
Women	890	67.1
Education		
Less than high school degree	35	2.7
High school	239	18.0
University	861	64.9
Postgraduate	191	14.4
Marital status		
Married	624	47.1
Single	702	52.9
Having children		
Yes	628	47.4
No	698	52.6
Income		
Low	500	37.7
Middle	408	30.8
High	418	31.5
Having chronic disease		
Yes	414	31.2
No	912	68.8
Following diet		
Yes	167	12.6
No	1159	87.4
Food allergy		
Yes	128	9.7
No	1198	90.3
Regular physical activity		
Yes	465	35.1
No	861	64.9
BMI group		
Underweight	88	6.6
Normal	670	50.5
Overweight	409	30.9
Obese	159	12.0
	*X* ± SD	
Age (years)	37.82 ± 14.07	
BMI (kg/m^2^)	24.63 ± 4.65	
Food label literacy	3.89 ± 1.94	
TOS–HeOr	14.70 ± 5.67	
TOS–OrNe	5.37 ± 4.66	

*BMI* Body Mass Index, *TOS–HeOR* Teruel Orthorexia Scale Healthy Orthorexia, *TOS–OrNe* Teruel Orthorexia Scale Orthorexia Nervosa

**Fig. 1:**
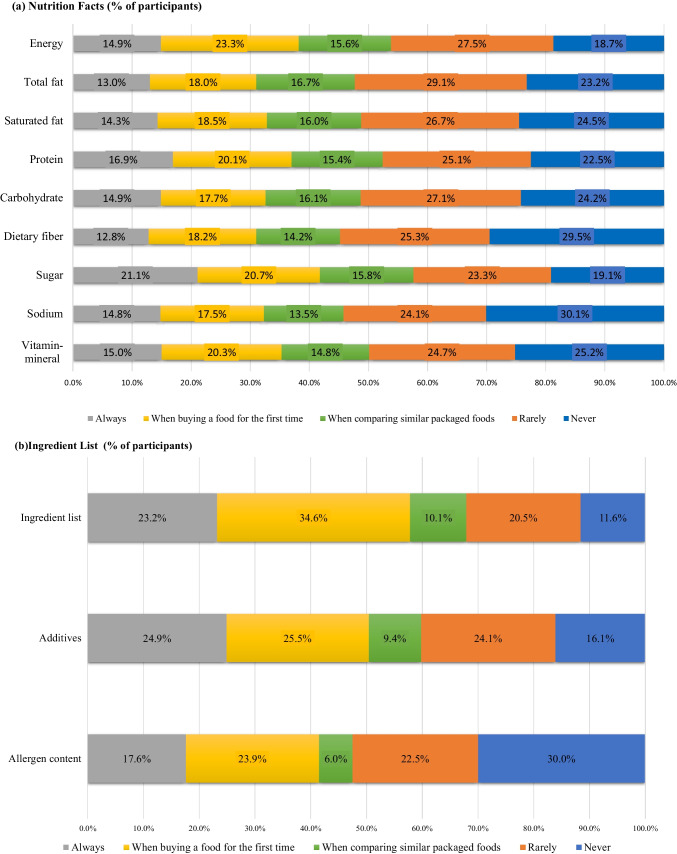

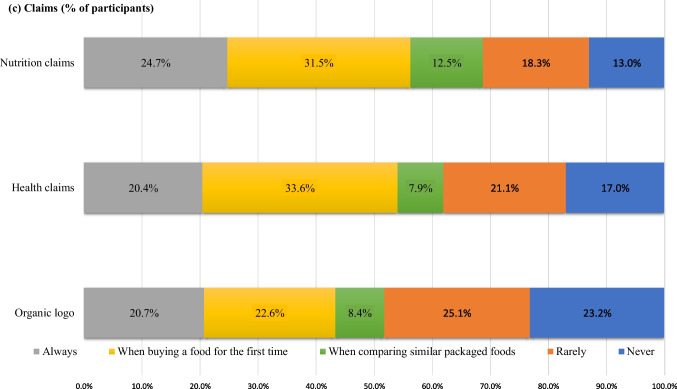
The distribution of the participants according to the frequency of use of the **a** nutrition facts, **b** ingredient list, and **c** claims

Figure 1 shows the frequency of use of the nutrition facts panel, ingredients list and claims. The proportion of participants who “always” read the items on the nutrition facts panel varied between 12.8% and 21.1% and who read the items “when comparing similar packaged foods” varied between 13.5% and 16.7%. Energy and sugar content were the most read sections, with only 18.7% and 19.1% of participants saying they “never” use these items. The least checked items were sodium, dietary fiber, and vitamin–mineral content, which were “never” read by 30.1%, 29.5% and 25.2% of the participants, respectively (Fig. 1a). The use of ingredients list and additives was widespread; specifically, 23.2% and 24.9% of the participants reported that they read these items “always”, and 34.6% and 25.5% of the participants reported reading them “when purchasing food for the first time” (Fig. 1b). Similarly, the majority of participants used nutrition and health claims, with only 13.0% and 17.0% of participants, respectively, stating that they 'never' use these items. Although 20.7% of the participants consistently checked the organic logo, 23.2% never used it (Fig. 1c).

Factors related to the use of food labels are shown in Table 2. After participants were classified as users and non-users based on their use of different sections of food labels it was found that 72.3% of them used the nutrition fact panel, 76.3% used the ingredients list, and 79.9% used the claims section of the food label. The age of the participants who read the ingredients list and the claims was higher compared to non-users (*p* < 0.05). Women were more likely to read all sections of the food label compared to men, including the nutrition facts panel (74.4% vs 68.1%, *p* = 0.017), the ingredients list (78.7% vs 71.6%, *p* = 0.004), and claims (84.3% vs 70.9%, *p* < 0.001). Educational level or self-reported income were not associated with food label use. However, there were significant differences according to marital and parental status. Married participants were more likely to read the ingredients list than singles (79.2% vs 73.8%, *p* = 0.021) and participants with children were more likely to read the ingredients list and claims than those without children (79.1% vs 73.8%, *p* = 0.022 and 82.5% vs 77.5%, *p* = 0.024, respectively). There were also significant differences in food label use by health-related variables such as having a chronic disease and following a diet. Participants with chronic diseases were more likely to utilize the ingredients list and claims sections compared to those who did not (80.2% vs 74.6%, *p* = 0.025 and 87.2% vs 76.5%, *p* < 0.001, respectively). Participants who follow a diet were more likely to use the nutrition facts panel and ingredients list sections compared to those who do not follow (83.8% vs 70.7%, *p* < 0.001, 85.6% vs 75.0%, *p* = 0.002, respectively). However, the proportions of the use of nutrition facts panel, ingredients list, and claims did not differ significantly by BMI or having a food allergy (*p* > 0.05). Among lifestyle habits, participants who exercise regularly were more likely to use all sections of the food label compared to those who did not (*p* < 0.05). Food label literacy was also associated with food label use. The nutrition facts panel, the ingredients list, and the claims users had higher food label literacy scores than non-user participants. Orthorexia scores also differed significantly between groups. TOS–HeOr scores were found higher among food label users compared to non-users and this trend was significant for all three sections of the food label (*p* < 0.05). Similarly, higher TOS–OrNe scores were obtained among the nutrition facts panel and ingredients list users compared to non-users (*p* < 0.05). However, there was no statistically significant difference in reading claims according to the TOS–OrNe scores.

**Table 2:** Associations of demographics, health status, lifestyle factors, and food label literacy with food label use among Turkish adults (*n* = 1326)

	Nutrition facts panel			Ingredients list			Claims		
Variable	User (*n* = 959)	Non-user (*n* = 367)	*p* value	User (*n* = 1012)	Non-user (*n* = 314)	*p* value	User (*n* = 1059)	Non-user (*n* = 267)	*p* value
Age (years) (X ± SD)	37.99 ± 14.46	37.36 ± 12.99	0.438	38.52 ± 14.20	35.55 ± 13.39	0.001*	38.59 ± 14.33	34.77 ± 12.53	< 0.001*
Sex									
Men	297 (68.1)	139 (31.9)	0.017*	312 (71.6)	124 (28.4)	0.004*	309 (70.9)	127 (29.1)	< 0.001*
Women	662 (74.4)	228 (25.6)		700 (78.7)	190 (21.3)		750 (84.3)	140 (15.7)	
Education									
Less than high school degree	23 (65.7)	12 (34.3)	0.653	24 (68.6)	11 (31.4)	0.598	30 (85.7)	5 (14.3)	0.839
High school	179 (74.9)	60 (25.1)		178 (74.5)	61 (25.5)		190 (79.5)	49 (20.5)	
University	620 (72.0)	241 (28.0)		663 (77.0)	198 (23.0)		688 (79.9)	173 (20.1)	
Postgraduate	137 (71.7)	54 (28.3)		147 (77.0)	44 (23.0)		151 (79.1)	40 (20.9)	
Marital status									
Married	445 (71.3)	179 (28.7)	0.439	494 (79.2)	130 (20.8)	0.021*	512 (82.1)	112 (17.9)	0.061
Single	514 (73.2)	188 (26.8)		518 (73.8)	184 (26.2)		547 (77.9)	155 (22.1)	
Having children									
Yes	446 (71.0)	182 (29.0)	0.314	497 (79.1)	131 (20.9)	0.022*	518 (82.5)	110 (17.5)	0.024*
No	513 (73.5)	185 (26.5)		515 (73.8)	183 (26.2)		541 (77.5)	157 (22.5)	
Income									
Low	368 (73.6)	132 (26.4)	0.684	386 (77.2)	114 (22.8)	0.208	405 (81.0)	95 (19.0)	0.344
Middle	294 (72.1)	114 (27.9)		299 (73.3)	109 (26.7)		316 (77.5)	92 (22.5)	
High	297 (71.1)	121 (28.9)		327 (78.2)	91 (21.8)		338 (80.9)	80 (19.1)	
Having chronic disease									
Yes	309 (74.6)	105 (25.4)	0.204	332 (80.2)	82 (19.8)	0.025*	361 (87.2)	53 (12.8)	< 0.001*
No	650 (71.3)	262 (28.7)		680 (74.6)	232 (25.4)		698 (76.5)	214 (23.5)	
Following diet									
Yes	140 (83.8)	27 (16.2)	< 0.001*	143 (85.6)	24 (14.4)	0.002*	142 (85.0)	25 (15.0)	0.075
No	819 (70.7)	340 (29.3)		869 (75.0)	290 (25.0)		917 (79.1)	242 (20.9)	
Food allergy									
Yes	92 (71.9)	36 (28.1)	0.905	105 (82.0)	23 (18.0)	0.110	107 (83.6)	21 (16.4)	0.268
No	867 (72.4)	331 (90.2)		907 (75.7)	291 (24.3)		952 (79.5)	246 (20.5)	
Regular physical activity									
Yes	370 (79.6)	95 (20.4)	< 0.001*	381 (81.9)	84 (18.1)	< 0.001*	393 (84.5)	72 (15.5)	0.002*
No	589 (68.4)	272 (31.6)		631 (73.3)	230 (26.7)		666 (77.4)	195 (22.6)	
BMI	24.50 ± 4.27	24.80 ± 4.88	0.300	24.56 ± 4.34	24.66 ± 4.77	0.735	25.00 ± 4.50	24.48 ± 4.43	0.080
Food label literacy	4.05 ± 1.86	3.49 ± 2.09	< 0.001*	3.99 ± 1.87	3.57 ± 2.12	0.002*	4.01 ± 1.87	3.41 ± 2.13	< 0.001*
TOS–HeOr (X ± SD)	15.15 ± 5.54	13.52 ± 5.85	< 0.001*	15.25 ± 5.50	12.90 ± 5.87	< 0.001*	15.12 ± 5.49	13.00 ± 6.05	< 0.001*
TOS–OrNe (X ± SD)	5.60 ± 4.60	4.77 ± 4.76	0.003*	5.51 ± 4.58	4.91 ± 4.87	0.045*	5.46 ± 4.63	5.02 ± 4.78	0.169

*BMI* Body Mass Index, *TOS–HeOR* Teruel Orthorexia Scale Healthy Orthorexia, *TOS–OrNe* Teruel Orthorexia Scale Orthorexia Nervosa. *p < 0.05. *p* value was calculated using Chi-square or student *t* test

The association (OR and 95% CI) of the orthorexia scores with the use of food labels are given in Table 3. The regression models were adjusted for potential confounding factors, including age, sex, marital status, having children, chronic disease, dieting, and physical activity in model 1. Food label literacy is additionally included in model 2. In the fully adjusted model, one point increase in the average TOS–HeOr scores (equal to a nine-point increase in total score) was associated with nearly 1.5 times the likelihood of reading the nutrition facts panel and 1.8 and 1.6 times the likelihood of reading the ingredients list and claims, respectively (*p* < 0.001). Similarly, one point increase in the average TOS–OrNe scores (equal to a seven-point increase in total score) was associated with a higher probability of reading the nutrition facts panel (OR 1.48, 95% CI 1.20–1.81), ingredients list (OR 1.27, 95% CI 1.03–1.57). The association between TOS–OrNe scores and reading claims was not significant in the unadjusted model and model 1. However, in the fully adjusted model, the increase in the TOS–OrNe scores was associated with reading claims section (OR 1.27, 95% CI 1.01–1.59).

**Table 3:** Logistic regression analysis evaluating the associations between food label use and orthorexia scores among Turkish adults (*n* = 1326)

	Nutrition facts panel		Ingredients list		Claims	
	OR (95% CI)	*p* value	OR (95% CI)	*p* value	OR (95% CI)	*p* value
*TOS–HeOr*^*a*^						
Unadjusted model	1.59 (1.31–1.93)	< 0.001*	1.96 (1.59–2.42)	< 0.001*	1.84 (1.47–2.29)	< 0.001*
Adjusted model^1^	1.48 (1.20–1.83)	< 0.001*	1.72 (1.38–2.16)	< 0.001*	1.58 (1.25–2.00)	< 0.001*
Adjusted model^2^	1.53 (1.24–1.89)	< 0.001*	1.76 (1.41–2.20)	< 0.001*	1.63 (1.29–2.07)	< 0.001*
*TOS–OrNe*^*a*^						
Unadjusted model	1.33 (1.10–1.61)	0.004*	1.23 (1.01–1.50)	0.045*	1.16 (0.94–1.43)	0.170
Adjusted model^1^	1.29 (1.06–1.57)	0.012*	1.14 (0.93–1.40)	0.195	1.10 (0.89–1.36)	0.393
Adjusted model^2^	1.48 (1.20–1.81)	< 0.001*	1.27 (1.03–1.57)	0.028*	1.27 (1.01–1.59)	0.040*

*CI* Confidence Interval, *TOS–HeOR* Teruel Orthorexia Scale Healthy Orthorexia, *TOS–OrNe* Teruel Orthorexia Scale Orthorexia Nervosa

^a^Indicates one point increase in the TOS–HeOR (9 item) and TOS–OrNe (7 item) averaged scores based on a 4 point Likert scale ranging from 0 = strongly disagree to 3 = strongly agree

* < 0.05 Adjusted model 1: Age, sex, marital status, having children, having chronic disease, following diet and physical activity. Adjusted model 2: Factors in the Model 1 plus food label literacy

## Discussion

To the best of our knowledge, the present study is the first to investigate the relationship between both aspects of orthorexia and reading different sections of food labels, including the nutrition facts panel, ingredients list, and claims. In particular, this study suggests that the sections of the food label that participants focused on more differed according to healthy orthorexia and orthorexia nervosa scores. Furthermore, specific sections of the food label were associated with different socio-demographic characteristics and health-related lifestyle habits.

Being healthy or healthy eating was identified by food label readers as the main reason for using food labels [23, 24, 38]. Using food labels has also been found to be positively associated with selecting foods for health reasons [19]. However, the obsessive thoughts about healthy eating would make a difference in the interest in food labels compared to non-pathological interest in healthy eating. On the other hand, the relationship between the use of food labels and orthorexia nervosa, has been investigated in only one study [26]. The study, which was conducted with 674 university students, found that students with a tendency towards orthorexia nervosa were more likely to read the energy, protein, carbohydrate, saturated fat, cholesterol, total fat, fiber, and sugar content on food labels compared to students without a tendency towards orthorexia nervosa. Furthermore, the study found that individuals who exhibited orthorexic tendencies were more likely to read and pay attention to various types of information on food labels, such as nutritional content, amount, health claims, usage instructions, food additives, nutritional values, and brand information, compared to individuals without such tendencies [26]. However, in the relevant study, food label reading behaviour was only evaluated concerning the pathological aspect of orthorexia and the scale that was used is insufficient to distinguish between healthy and pathological orthorexic behaviour. Therefore, it remained unclear whether this attitude is related to healthy and/or pathological aspect of orthorexia. Unlike Yardımcı et al. [26] the current study evaluated the orthorexic tendency both in healthy and pathological aspects to evaluate whether there is a difference on food label reading. Our results indicated that both aspects of orthorexia were related to food label use. Higher healthy orthorexia scores were associated with reading all three sections of a food label. Besides, higher orthorexia nervosa scores were associated with reading nutrition fact panel and ingredients list in the univariate analysis. Although orthorexia nervosa scores did not differ in terms of using claims section, when the all-potential confounders included to the regression model it became significantly associated with usage of claims. More importantly, the current study revealed that the healthy aspect of orthorexia demonstrated the highest association with reading the ingredients list (OR 1.76, 95% CI 1.41–2.20), while the TOS–OrNe scores showed the strongest association with reading the nutritional facts panel (OR 1.48, 95% CI 1.20–1.81). Accordingly, it could be suggested that the most focused section of the food label may differ depending on the aspect of orthorexic traits. It can be hypothesized that individuals with higher TOS–OrNe scores could have focused more on the nutrient quantities provided in the nutrition facts panel, which is particularly notable given that orthorexia nervosa is a type of eating disorder. Conversely, those with higher TOS–HeOr scores may have focused more on the ingredients section, as it provides information about the sources of these nutrients and amounts of each ingredient. As a different point of view, food label use and food label knowledge may also affect orthorexic tendencies. Therefore, it should be considered that the interaction may be bi-directional. Since, there is no other research focused on different parts of the food label and different aspects of orthorexia, these hypotheses should be further examined and tested by additional research. Furthermore, conducting studies to explore the motivations behind reading various sections of food labels in relation to orthorexic tendencies will contribute to a more comprehensive understanding of the issue.

Studies on food label use typically focus on overall food label reading habits despite the different types of information provided by nutrition facts, ingredients list, and health claims [11–13, 15–19]. Limited studies have evaluated the use of different sections of the food label with conflicting results. The prevalence of participants reading different parts of the food label ranged from 43.8% to 61.5% in the NHANES study [22]. Food Drug Administration (FDA)’s Food Safety and Nutrition Survey (FSANS) showed that 87% of participants read nutrition facts panel [39]. On the contrary, Cristopher et al. found that only 30% of consumers use nutrition facts panel and approximately 65% of those read the ingredients list [11]. In this study, the prevalence of participants reading different parts of the food label were 72.3% for nutrition fact panel, 76.3% for ingredients list and 79.9% for claims which is generally higher compared to NHANES [22] and Cristopher et al.’s study [11] and lower compared to FSANS [39]. It is worth noting that in the FSANS [39], participants were asked whether they ever look at the nutrition facts label, whereas other studies including ours questioned a repetitive label use behaviour. The higher prevalence of food label usage in our results compared to NHANES [22] and Cristopher et al.’s study [11] could be attributed to various factors, such as methodological difference in the classification of the food label user, and variations in the sociodemographic characteristics of the study samples. In our study, the percentage of female and highly educated participants were higher compared to Cristopher et al.’s study [11]. The distribution was balanced between income classes in our study while over 80% of individuals had low or middle income in previous study. These characteristics were mentioned above as contributing factors for food label using behaviour [11–13]. Also, there are several other factors affecting the use of food labels. Food label literacy is one of the major factors indicating consumers to understand the information and compare different products [15, 21]. Unfortunately, there is no information about food label literacy in these studies, we failed to compare the results in terms of food label literacy levels. More importantly, the higher prevalence of food label reading may also be related to the fact that the data were collected during the COVID-19 period. Several studies indicated a differed food choice attitude due to different motives in the pandemic period. In fact, studies have reported that consumer interest in food labels increased during the pandemic [40], and the fear of COVID-19 was associated with an increase in reading food label [41].

When considering the information presented on the nutrition facts panel, the results of this study align with prior research [11, 42] showing that energy and sugar content are the most read sections, while sodium, dietary fiber, and vitamin–minerals are fewer read sections. FDA’s FSANS report indicated that sodium along with energy and total sugar content is among the top four items that consumers read which is different from our study in terms of sodium content [39]. Given the fact that the high sodium consumption (men:4806 mg/d and women:3693 mg/d) in Türkiye, the lack of attention to sodium content was considered a situation that should be addressed [43]. Claims and ingredients list of the food labels were used by the majority of participants, and they were mostly used “when buying the food for the first time” in the current study. These results indicate that claims and ingredients list are used by consumers to have information about new food; suggesting that these parts can be more understandable compared to the nutrition facts panel.

Prior research indicates that factors such as being female [11–14, 16, 21–23], having higher level of education [11–14, 16, 22, 44–46], and income [11, 13, 16, 22, 44, 46] were associated with more likely to use food labels. Consistent with the literature this study demonstrates that women were found to be more likely to read all three sections of food labels. This finding may be attributed to several factors, including higher levels of nutrition knowledge [12, 16], greater awareness of the importance of reading food labels [12], and the higher prevalence of dieting and eating disorders [47] among women. In addition, the fact that grocery shopping is usually done by women [48] it may be a possible explanation for this result, as those responsible for grocery shopping were found to be more likely to be users of food labels [21]. Furthermore, having children was associated with using the ingredients list and claims sections of food labels. This finding supports studies suggesting that parents check food labels to prevent their children from consuming unhealthy foods [49] and that nutrient claims have a stronger impact on mothers' perception of health and snack choices for their children compared to the nutrient content [50]. The findings from studies investigating the relationship between age and food label use are inconsistent [11, 12, 14, 17, 21–23]. In this study, using the ingredients list and claims were found to be related to older age, but reading the nutrition facts was not related.

The use of food labels is associated with various health-related lifestyle habits. Having specific weight goals [11], diet awareness [17], and being physically active [11, 23] were found to be more likely to lead to using food labels and nutrition fact panels. In line with these findings, the present study found that regular physical activity was associated with the likelihood of reading all sections of the food label, and dieting was related to using a nutrition facts panel and ingredients lists. These findings were not surprising, given that diet and exercise are known to increase individuals' interest in learning about the content and nutritional value of the foods they consume [51]. In this study BMI was not related to food label use. Although, Storz [14] reported that individuals who sometimes read food labels had a significantly higher BMI than those who rarely read them, other studies indicated that there was no association between using food labels and BMI [12, 42]. It should be noted that in our study BMI was calculated by self-reported weight and height measurements and the data was mostly obtained during COVID-19 pandemic which could lead people to use food labels more often. These factors may have supressed the potential effects of BMI on food label use.

The relationship between reading food labels and having a chronic disease varies across studies. Some studies have found that individuals with chronic diseases have lower usage of food labels [46], while others have found that they use them more frequently [12, 17], and one has found no relationship [44]. These conflicting results may be due to different reasons that need to be evaluated in more depth, including the disease itself, its severity and disease onset time. Individuals tend to switch on a healthier diet by changing their food choices and nutrient intake after the diagnosis of chronic diseases [52–54]. This study showed that reading the ingredients and claims section was associated with having a chronic disease, while reading nutrition facts was not associated. Individuals with chronic diseases are often exposed to messages from health counsellors to avoid foods high in fat and sugar and to choose products with reduced fat, sugar and sodium. This may be associated with increased scrutiny of the claims section of the food label, which includes nutrition claims such as "fat free", "sugar free" and "low sodium", and health claims that provide a statement about a relationship between a food and health. The level of understanding of food labels also plays a decisive role in the impact of food labels on food purchasing behaviour [15]. Rashaideh et al. found that consumers with higher food label knowledge were more likely to use them [21]. Specifically, this study revealed that higher food label literacy was linked to an increased likelihood of using all sections of food labels. Therefore, having sufficient food label literacy appear to be critical factor for making healthy food choices.

### Strengths and limitations

The main strength of this study is reporting, for the first time, the associations between the usage of different sections of food labels and both pathological and healthy aspects of orthorexia. However, there are some limitations of the study. Firstly, it is important to note that the data were mostly collected during the COVID-19 pandemic. Hence, the likely impact of the pandemic on individuals' food label reading and health-related behaviours must be considered when interpreting the results. Secondly, it is possible that the study design, which involved an online survey with a snowball sampling method, may have resulted in a selection bias among internet users. However, according to the Turkish Statistical Institute, in 2021, the rate of households having access to the Internet from home was 92.0%, and the percentage of individuals using the Internet in the 16–74 age group was 82.6% [55]. In addition, in this study, participants were mainly recruited through WhatsApp and Instagram. These statistics are in line with social media usage in Türkiye. The most used social media and messaging applications among individuals in Türkiye were WhatsApp (82.0%), YouTube (67.2%), and Instagram (57.6%) [56]. Thirdly, there is a potential for social desirability bias in self-reported measures of food label use, food label literacy, and self-reported data on weight and height used to calculate BMI. In addition, it should be noted that only the food label literacy sub-dimension of the EINLA scale was utilized which needs further investigation to comprehensively understand the relationship between food label literacy, food label use and orthorexia. Future studies may therefore assess nutritional literacy using all parts of the EINLA scale. Finally, it is important to note that the orthorexic tendency was evaluated using scale, and the scores do not represent a clinical diagnosis due to the lack of official diagnostic criteria for orthorexia nervosa. Therefore, caution is needed in interpreting the study's findings related to orthorexia tendencies.

### What is already known about this subject?

It is known that reading food labels is associated with various factors such as age, gender, diet, and nutrition and food label knowledge. Moreover, a recent study revealed that university students with higher orthorexic tendencies were more likely to read food labels. However, it remains unclear whether there is a relationship between food label use and the pathological and healthy aspects of orthorexia, and whether food label literacy is associated with reading specific sections of the food label.

### What does this study add?

This study is unique in its ability to present data on the prevalence of using the nutrition facts panel, the ingredients list, and the claims section of food labels, as well as associated factors with the use different parts of food label including food label literacy, orthorexic tendencies (healthy orthorexia and orthorexia nervosa), and socio-demographic and health-related factors among adults.

## Conclusion

This is the first study to examine the relationship between the use of different sections of food labels and healthy and pathological orthorexia tendency in adults. The study demonstrates that food label users have higher orthorexia scores (healthy and pathological) compared to non-users. In addition, it has been proposed that the most focused part of the food label may vary depending on the aspects of orthorexia. However, further research is needed to investigate this hypothesis.

### Supplementary Information

Below is the link to the electronic supplementary material.

Supplementary file1 (PDF 70 KB)

## Data Availability

The data sets generated during and/or analysed during the current study are available from the corresponding author on reasonable request.
