# Supplementary figures and images for: Factors associated with food label use: focus on healthy aspects of orthorexia and orthorexia nervosa
Source: Eat Weight Disord. 2024 May 4;29(1):32. doi: 10.1007/s40519-024-01661-9 (PMC11069476; doi:10.1007/s40519-024-01661-9)

Supplementary Figure 1.

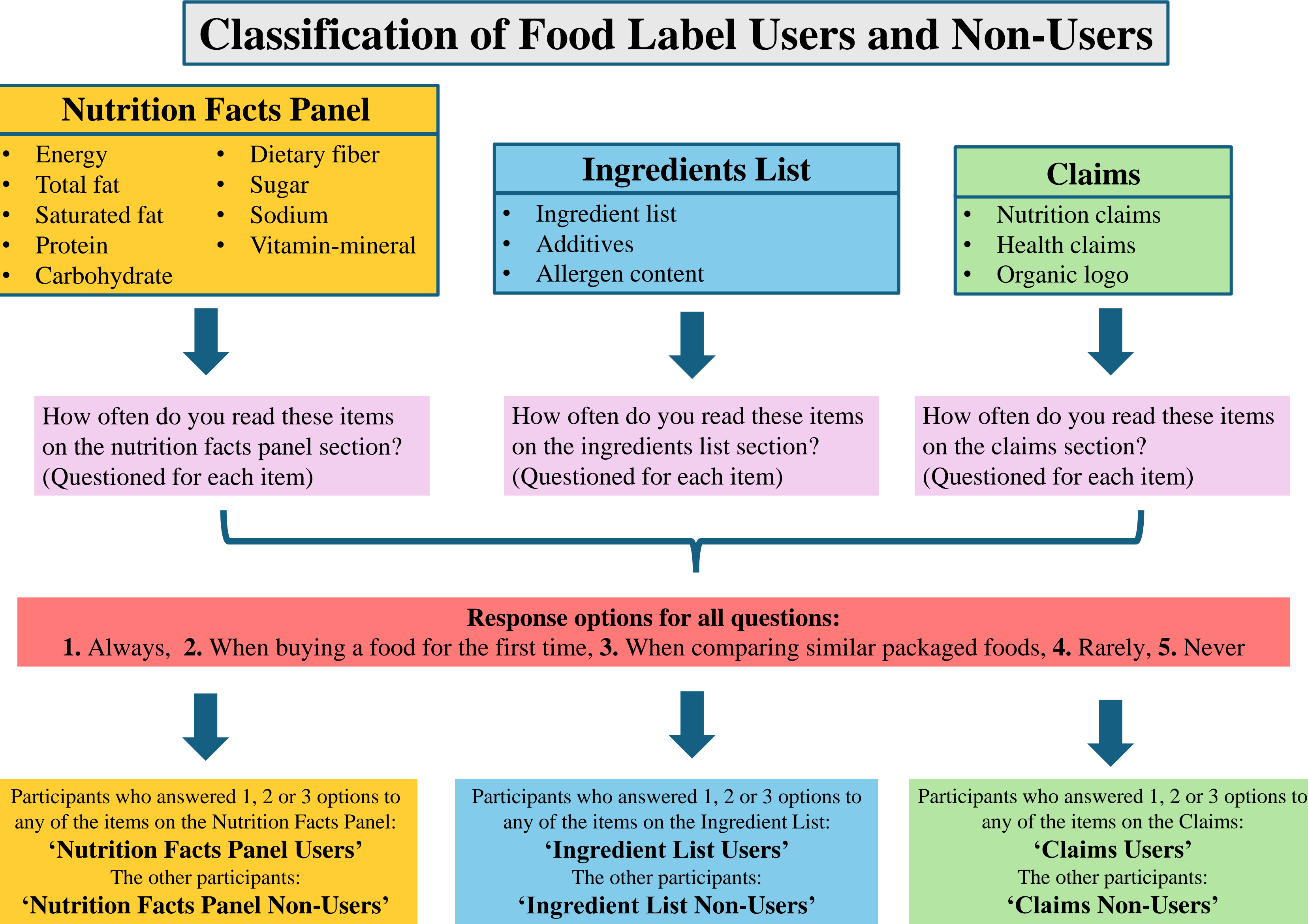

Supplement: Supplementary file 1 — Supplementary file1 (PDF 70 KB) [file 40519_2024_1661_MOESM1_ESM.pdf]
